# Risk stratification of hypertension in South Africa: a systematic review with meta-analysis

**DOI:** 10.3389/fcvm.2026.1710798

**Published:** 2026-02-23

**Authors:** Martins Nweke, Nalini Govender, Julian Pillay

**Affiliations:** 1 Global Health and Sustainability, Faculty of Health Sciences, Durban University of Technology, Durban, South Africa; 2 Basic Medical Sciences, Faculty of Health Sciences, Durban University of Technology, Durban, South Africa

**Keywords:** determinants, hypertension, risk factors, South Africa, stratification

## Abstract

**Background:**

Hypertension is a leading cause of morbidity and mortality in South Africa. Evidence on risk factors is scattered across study designs, limiting clinical decision support. This review synthesizes evidence to classify hypertension risk factors to strengthen prediction models and prevention strategies through causal integration.

**Methods:**

A systematic review followed PRISMA 2020, searching PubMed, Scopus, Web of Science, Medline, CINAHL, and SABINET. Two independent reviewers screened and extracted data. Narrative synthesis and meta-analysis were performed, and risk factors were classified within a framework. Causal pathways were mapped using directed acyclic diagram.

**Results:**

Eleven studies with 49,058 participants from nine provinces were included. Strongest risk factors were advanced age (OR: 3.67, CI 1.7–7.6) and diabetes (OR: 1.85, CI 1.39–2.46), followed by low education (OR: 1.75, CI 1.56–2.00), ethnicity (OR: 1.57, CI 1.23–2.01), and socioeconomic status (OR: 1.14, CI 1.01–1.30). Critical risk thresholds (Rw: 3.8 and 5.5) correspond to the 75th and 50th percentiles. A prediction model (Age–Ethnicity–Diabetes) achieved Rw 6.0 and net GTT 2.49. Higher education, improved socio-economic status, and diabetes management were primary prevention strategies (Rw: 4.71, net GTT: 2.49). Age and diabetes emerged as “necessary causes”.

**Conclusion:**

Older adults (≥50 years), individuals with diabetes, mixed-race ethnicity, and lower socioeconomic status are priority groups. Screening should be prompted by age and diabetes.

**Registration:**

https://www.crd.york.ac.uk/PROSPERO/view/CRD420251026501, PROSPERO ID: CRD420251026501.

## 1 Introduction

Hypertension represents a significant global health challenge, contributing to the onset of cardiovascular diseases, renal complications, and premature mortality (1). Between 1990 and 2019, the prevalence of hypertension among adults doubled, with approximately one-third of the adult population diagnosed in 2019 (2). This prevalence is notably higher in low- and middle-income countries, of which 78% are adults (2). In Africa, the prevalence of hypertension is approximately 30.8%, with awareness and control rates lower than those in high-income countries (3). In South Africa, about 48.2% of adults had hypertension in 2016, with one-third remaining unaware of their condition (4). Although rapid urbanization and behavioral risk factors have been linked to this increasing prevalence (5), existing evidence on the determinants of hypertension and their relative significance remains fragmented in this region, exhibiting considerable spatial variation. Numerous studies have identified key determinants of hypertension in South Africa (6–9); however, a comprehensive systematic profiling of these determinants and their relative importance across different contexts and subpopulations is lacking.

The variability in the ranking of hypertension determinants is considerable and may be attributed to an overemphasis on statistical significance, potentially neglecting other causal elements. Current profiles of hypertension risk factors predominantly rely on statistical significance (6–11). In a national cohort survey conducted in South Africa, only six factors were significantly associated with hypertension (6), whereas other studies within the country have identified different risk factors as most prominent [7-1]. Wand et al. (11) identified obesity and elevated waist circumference as the primary risk factors for hypertension in a national cohort study. Conversely, data from a provincial study conducted by Ntuli et al. (8), reported age, marital status, and level of education as significant correlates of hypertension. The stratification of hypertension risk factors is complex and should incorporate factors beyond statistical significance, such as ethnic/geographic differences, temporal factors, and study designs.

Efforts to mitigate the risk factors associated with hypertension are considerably hindered by its polygenic and complex nature, similar to other noncommunicable diseases (12). Prioritizing significant risk factors through comprehensive approaches, such as lifestyle modifications, screening, early identification, community health promotion, and timely treatment, represents an effective and sustainable strategy (13). However, a comprehensive and rigorous framework that transcends statistical inference and incorporates robust epidemiological models of disease causation is essential for identifying key risk factors (14). A systematic review will facilitate a structured synthesis across diverse populations, methodologies, and contexts, thereby establishing a robust foundation to inform evidence-based and tailored prevention strategies. Consequently, this study aims to characterize and stratify the risk of hypertension in South Africa (SA) through a rigorous and systematic analysis.

### 1.1 Review questions

To achieve the study objective, we asked the following questions:

What are the risk factors of hypertension in SA?
What are their ranks when stratified via a nuanced stratification framework?
What is the critical risk threshold for predicting hypertension in SA?
What are the causal paths for hypertension in SA?

## 2 Conceptual and theoretical underpinning

To explore the complex risk factors for hypertension in South Africa, we utilized a nuanced framework: Rothman causal pie and Nweke's cumulative risk framework (15). This model was chosen for their ability to comprehensively capture the intricacies of disease causation and to overcome the limitations of individual theories (15). These epidemiological frameworks played a crucial role in directing both the data extraction and risk stratification processes in this systematic review. The epidemiological triangle, in particular, allowed for an extensive sampling of risk factors, providing a detailed understanding of the biological, personal and environmental factors. The Nweke's cumulative risk index improved the estimation of cumulative risk by integrating Hill's criteria and Rothman's model (15–20).

## 3 Methods

### 3.1 Protocol and registration

To ensure transparency, reproducibility, and methodological rigor, this systematic review adhered to the Preferred Reporting Items for Systematic Reviews and Meta-Analyses (PRISMA) protocols (21). A checklist for systematic reviews of risk and etiology was employed (14, 15). The review protocol was registered with the International Prospective Systematic Review Register (PROSPERO) under the identification number CRD420251026501. By following these recommendations, a systematic approach to study selection, data extraction, risk of bias assessment, evidence synthesis, and risk stratification was ensured. The protocol specified the inclusion and exclusion criteria, thereby ensuring methodological consistency. In addition, this study adhered to the guidelines for reporting observational data from included studies, as outlined in the Strengthening the Reporting of Observational Studies in Epidemiology guidelines, where applicable. This strategy ensured the preservation of the methodological integrity of the reviewed studies.

### 3.2 Eligibility criteria

The PECODTL framework, which encompasses population, exposure, comparison, outcome, design, timeframe, and language, was employed to ensure a systematic approach to defining the eligibility criteria for this systematic review. This framework enhanced the clarity of study selection and facilitated comparability across a diverse range of eligible studies.

#### 3.2.1 Inclusion criteria

##### 3.2.1.1 Population (P)

The population comprised studies conducted in South Africa that involved individuals with or without hypertension, as observed in case-control or cohort studies, irrespective of age or sex. The inclusion of both hypertensive and non-hypertensive individuals facilitated a comparative analysis aimed at identifying risk factors, as is typical in case-control or cohort studies.

##### 3.2.1.2 Exposure (E)

This review considered four categories of exposure: biological agents (e.g., obesity, diabetes, obstructive sleep apnea, kidney disease, use of nonsteroidal anti-inflammatory drugs, thyroid and adrenal disorders, and HIV-related factors); behavioral factors (e.g., smoking, consumption of table salt, alcohol, red meat, sugar-sweetened beverages, low intake of green vegetables, sedentary lifestyle, and negative emotions such as anger, anxiety, and sadness); psychosocial factors (e.g., stress, depression, loneliness, age, sex, income, education level, healthcare access, occupation, marital status, homelessness, and ethnicity); and environmental exposures (e.g., carbon monoxide, ozone, nitrogen dioxide, sulfur dioxide, heavy metals, and particulate matter).

##### 3.2.1.3 Comparison (C)

Comparative analyses were conducted between individuals with hypertension and those without. In addition, studies that provided risk assessments by comparing different levels of exposure, such as people living with HIV (PLWH) undergoing antiretroviral therapy (ART) vs. those who were ART-naïve, were incorporated**.**

##### 3.2.1.4 Outcome (O)

The primary outcome was the incidence or prevalence of hypertension, defined using the following criteria: systolic hypertension, characterized by a persistent elevation of systolic blood pressure (BP) ≥130 mmHg (22); diastolic hypertension, defined as a persistent elevation of diastolic BP ≥90 mmHg (22); a documented medical history of hypertension; or current use of antihypertensive medication.

##### 3.2.1.5 Design (D)

Case-control and cohort studies were included. Identifying risk factors required an appropriate comparison group to ascertain the relationship between exposure and outcome. Although cohort studies were regarded as the gold standard, case-control studies served as close approximations, particularly when the outcome was rare (23). Although hypertension was not an uncommon outcome in South Africa, the estimation of temporality based on study design permitted the integration of both case-control and cohort designs to identify risk factors and estimate their corresponding risk attributions (15).

##### 3.2.1.6 Timeframe (T)

This review encompassed longitudinal studies, including cohort and case-control studies, conducted from database inception until May 2024. To ensure a robust temporal association between exposure and the risk of hypertension, only studies with clearly defined follow-up periods were included.

##### 3.2.1.7 Language

Articles composed in languages other than English, such as IsiZulu and Afrikaans, were translated using Google Translate.

#### 3.2.2 Exclusion criteria

Studies that investigated disease conditions other than hypertension, where no separate analysis was conducted, were excluded. Studies were also excluded if they explored biological or nonbiological variables without assessing their contribution to hypertension, if predictors and outcomes could not be distinguished, or if they reported only prevalence or frequency of risk factors without measures of association such as odds ratios (ORs) or hazard ratios (HRs). In addition, studies were excluded if hypertension was not the primary outcome or if they failed to provide risk estimates for hypertension. With respect to study design, simple cross-sectional studies that did not establish temporality, as well as qualitative studies, editorials, case reports, conference abstracts, and commentaries, were excluded**.**

### 3.3 Information sources

A comprehensive search of electronic databases was conducted to ensure that all relevant studies were retrieved. The search covered PubMed/MEDLINE, SCOPUS, EMBASE, the Cochrane Library, Web of Science, the Cumulative Index for Nursing and Allied Health Literature (CINAHL), and African Journals Online (AJOL), from their inception to May 2024. In addition, the reference lists of all included studies and relevant systematic reviews were manually screened to identify further eligible articles. To minimise publication bias, grey literature sources, including conference proceedings and preprint repositories, were also considered. Where full texts of potentially eligible articles were unavailable, study authors were contacted directly to obtain the necessary information.

### 3.4 Search strategy

An information specialist created the search strategy. The search strategy utilised Medical Subject Headings in combination with free-text keywords. The initial search was conducted in PubMed and subsequently was adapted for each database using appropriate Boolean operators (AND, OR, NOT) and truncation symbols, as required. The strategy was further adapted for other databases using database-specific syntax (Supplementary File 1).

*PubMed search strategy:* (“Hypertension” OR “High blood pressure” OR “metabolic syndrome” OR “cardiometabolic disease”) AND (“Risk Factors” OR “Correlates” OR “Associated factors” OR “Factor associated with” OR “Comorbidities” OR “Epidemiology” OR “Causality” OR “Predictors”) AND “SSA” OR “Africa”.

### 3.5 Data management and selection of studies

The retrieved studies were imported into EndNote 21 to facilitate the removal of duplicates and the organisation of references. Study selection followed a two-step screening process to ensure methodological rigour and reduce bias. Two independent reviewers screened the studies, and disagreements were resolved through discussion or in consultation with a third reviewer.

First, titles and abstracts were assessed on the basis of the established inclusion and exclusion criteria. Eligible studies, or those that lacked sufficient information for a definitive decision, proceeded to full-text review. In the second step, full-text articles were obtained and assessed for final inclusion. Reasons for exclusion were systematically documented**.**

**Figure 1:**
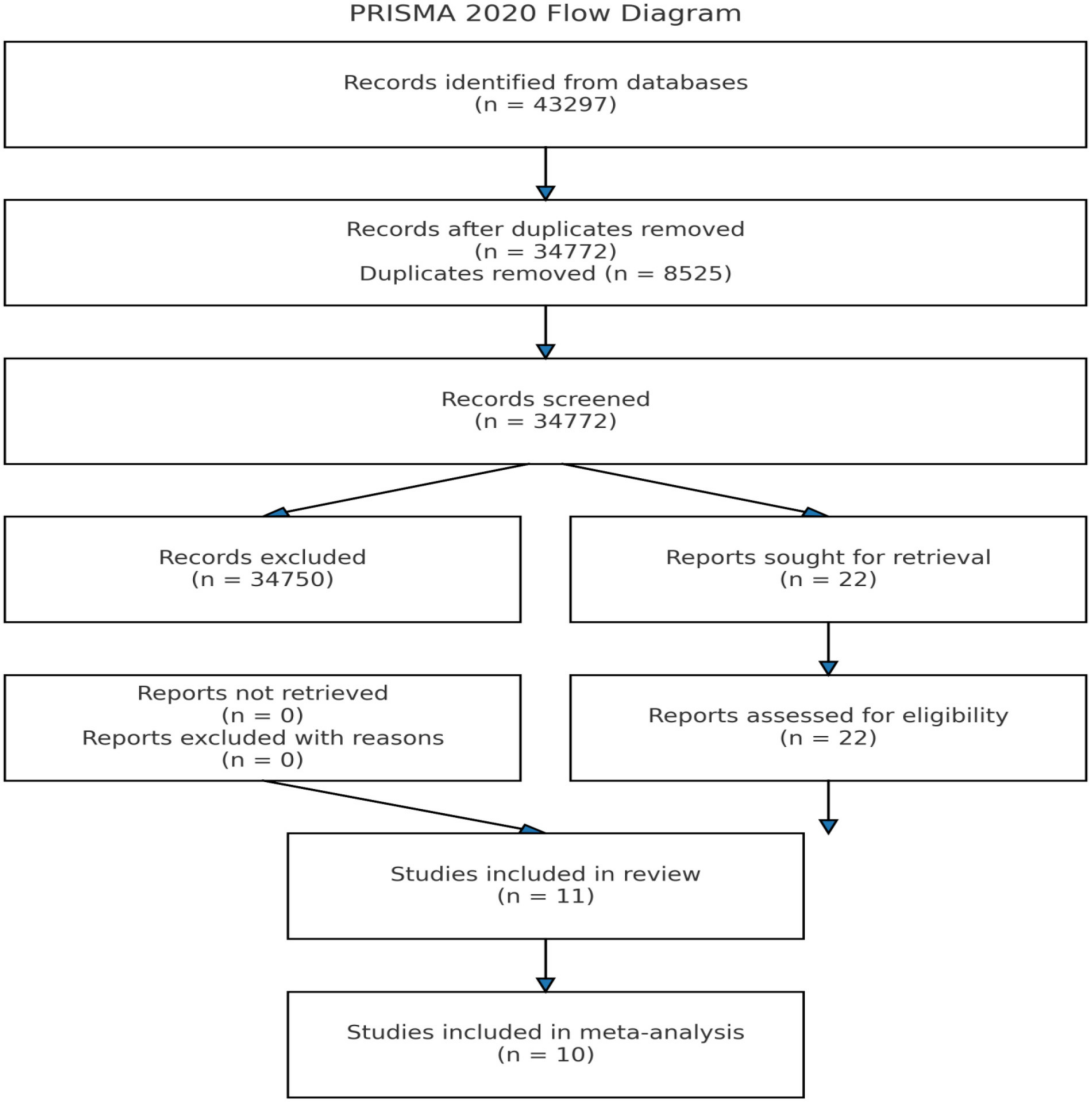
PRISMA flow diagram for the systematic review of hypertension risk factors in South Africa.

The selection process was depicted using a PRISMA flow diagram (Figure 1), illustrating the number of records identified, screened, included, and excluded, along with reasons for exclusion. From the outset, the independent reviewers agreed on timelines, and the principal reviewer tracked the screening process through phone calls and text messages.

### 3.6 Data collection

#### 3.6.1 Data extraction

A standardised data extraction template was used to record study details, exposure variables, and outcome metrics. Two reviewers independently extracted the data, and disagreements were resolved through discussion. The principal reviewer tracked the data extraction through phone calls and text messages. From the outset, the independent reviewers agreed on timelines. The data items were extracted as follows.

#### 3.6.2 Data items

##### 3.6.2.1 Study characteristics

Study characteristics included the authors' names, year of publication, study location, study design, number of participants, and participant demographics. Demographic information comprised age, sex, HIV status, and any comorbid conditions.

##### 3.6.2.2 Exposure variables

Exposure variables were categorised into four domains. Biological factors included obesity, diabetes, use of anti-inflammatory drugs, thyroid and adrenal disorders, congenital heart defects, HIV infection, and antiretroviral therapy. Behavioural factors included smoking, discretionary salt intake, alcohol consumption, red meat consumption, low intake of green vegetables, physical inactivity, and high consumption of sugar-sweetened beverages. Personal factors encompassed age, sex, income, education level, access to healthcare, occupation, marital status, homelessness, loneliness, ethnicity, emotional states, and stress. Finally, environmental factors included exposure to ambient air pollutants, cold temperatures, high altitudes, loud noise, heavy metals, organic solvents, and pesticides.

##### 3.6.2.3 Effect (risk) measures

The frequency of each risk factor, hypertension risk estimates (e.g., OR, RR, and HR) with corresponding confidence intervals, and the statistical models used for risk adjustment were extracted.

#### 3.6.3 Outcome variables

##### 3.6.3.1 Primary outcome

Hypertension was defined using established thresholds: resting office blood pressure ≥130/80 mmHg, daytime ambulatory blood pressure ≥135/85 mmHg, or office blood pressure ≥140/90 mmHg, as well as a documented history of hypertension (22–24). Risk estimates (strength of association) described the degree to which two variables were related, reflecting both the magnitude and significance of their relationship (14, 15). Common measures of association included the odds ratio (OR), relative risk (RR), and hazard ratio (HR).

##### 3.6.3.2 Secondary outcomes

These included study design, data collection approach (prospective or retrospective), follow-up duration, sample size, sampling technique, study setting, and statistical models (univariable or multivariable).

##### 3.6.3.3 Derived outcomes

Critical risk threshold: This concept was derived from the stratification of identified risk factors and represented the point at which the risk of a particular outcome, such as hypertension, became evident, necessitating the implementation of preventive measures. The CRT was generally defined between the 75th and 100th percentiles of the cumulative risk weight in strategies targeting high-risk interventions. The selected percentile was based on the assumption that it produced a sufficient outcome (15, 18). The critical risk point guided interventions or preventive actions by identifying the risk levels that required attention (18).

###### 3.6.3.3.1 Causality elements

Temporality is central to causality, requiring that exposure occurs before the outcome. Cohort and experimental studies were valued because they established exposure–outcome time-sequence relationships (25). Consistency reflected reproducibility across populations, study designs, and methods, measured by the proportion of significant associations identified in systematic reviews. Irreversibility strengthened causal inference when effect directions remained stable across studies. The causality index quantified the relative contribution of each factor compared with others. Public health importance integrated this index with the stage and nature of a factor, recognising that factors with lower causality scores could, in some contexts, outweigh stronger determinants (20, 26).

#### 3.6.4 Exposure definition

In eligible studies, exposure variables were defined using standard clinical thresholds. Hypertension was considered present if systolic blood pressure was ≥140 mmHg, diastolic blood pressure was ≥90 mmHg, antihypertensive medications were being used, or there was a documented history of hypertension. Diabetes was defined as fasting glucose ≥126 mg/dl or HbA1c ≥6.5%. Dyslipidaemia was identified when low-density lipoprotein (LDL) cholesterol was ≥130 mg/dl or total cholesterol was ≥200 mg/dl. Hyperhomocysteinaemia was defined as serum homocysteine ≥9.45 μmol/L, and hyperuricaemia was defined as uric acid ≥7.0 mg/dl in men or ≥6.0 mg/dl in women. Obesity was defined as a body mass index (BMI) ≥30 kg/m^2^. Smoking referred to current or past tobacco use confirmed by self-report or biochemical validation, while alcohol use referred to current or past consumption assessed through self-report or biochemical validation. Other exposures were defined according to standard, validated measures.

For each eligible study, reported risk estimates (odds ratios, relative risks, or hazard ratios) were extracted and, where necessary, were converted into relative risks. Studies that did not directly report these measures but provided sufficient raw data to allow calculation were also included in the analysis.

### 3.7 Risk of bias assessment

#### 3.7.1 Assessment tool and process

Methodological quality was assessed using the Joanna Briggs Institute (JBI) risk-of-bias assessment tool, which was tailored for case–control and cohort studies (27). This tool provided a structured evaluation of study design, sampling, measurement, and analytical techniques to determine the potential risk of bias in each study.

#### 3.7.2 Domains of bias

The risk of bias assessment addressed several key methodological domains. Selection bias was evaluated by examining whether study participants were clearly defined, appropriately represented the target population, and were selected in ways that minimised bias. Information bias was assessed by reviewing the accuracy and reliability of exposure and outcome measurements, with particular attention to the use of standardised or validated tools. Confounding bias was considered in terms of whether studies adequately accounted for potential confounding variables through their design or statistical methods. Follow-up and attrition bias were analysed in cohort studies to determine whether follow-up was sufficient and whether any losses to follow-up were reported and appropriately managed. Finally, reporting bias was assessed by examining the potential for selective reporting of outcomes and ensuring that all relevant risk factors were transparently presented.

#### 3.7.3 Risk of bias classification

Each study was assessed and classified according to the Joanna Briggs Institute (JBI) criteria as having a low, moderate, or high risk of bias. Methodological limitations were considered in terms of their potential to affect the validity of study findings.

Low risk of bias: There was little or no concern that methodological issues compromised the validity of the findings.
Moderate risk of bias: Some methodological concerns were identified**,** but these were unlikely to materially affect the study conclusions.
High risk of bias: Significant methodological limitations were present and were likely to substantially affect the validity of the study outcomes.

### 3.8 Effect measures

#### 3.8.1 Primary summary measures

The relationships between various exposures and hypertension were evaluated using odds ratios (ORs), relative risks (RRs), or hazard ratios (HRs). Effect sizes were collected from the included studies to gauge the magnitude of hypertension risk associated with different exposures. In addition, 95% confidence intervals (CIs) were reported to indicate the precision of each estimate.

#### 3.8.2 Effect size interpretation

Effect measures (ORs, RRs, and HRs) were standardised. Values greater than 1.0 indicated increased hypertension risk, values less than 1.0 suggested a protective association, and a value of 1.0 indicated no association. Small effects were defined as 1.1–1.5, moderate effects as 1.6–2.5, and large effects as greater than 2.5. For example, an OR of 1.3 reflected a small increase in risk, an OR of 2.0 reflected a moderate increase, and an OR greater than 3.0 indicated a strong association. These thresholds followed established epidemiological conventions and GRADE guidance (28, 29).

### 3.9 Data synthesis and analysis

#### 3.9.1 Basic synthesis (narrative synthesis, meta-analysis, heterogeneity, sensitivity and publication bias)

A narrative synthesis examined associations between hypertension and identified risk factors, with study findings tabulated for clarity. Meta-analyses were conducted using random-effects models to account for both within-study and between-study variability, given the expected clinical and methodological heterogeneity across included studies. We used a complete case analytical approach that included only studies reporting relevant data. Pooled relative risks (RRs) and corresponding 95% confidence intervals were estimated using the inverse variance method. Where appropriate, risk estimates were converted into relative risks (RRs), with 1/RR applied to values less than 1 to standardised direction, and confidence intervals were adjusted using [1/OR^2^]. Analyses were performed using R version 4.3, with α set at 0.05.

Statistical heterogeneity was assessed using the I^2^ statistic. Values of approximately 25%, 50%, and 75% were interpreted as low, moderate, and high heterogeneity, respectively. Sensitivity analyses were not performed because several pooled analyses were based on a limited number of studies, and exclusion of individual studies would have materially reduced statistical power and stability of the pooled estimates. Consequently, findings were interpreted with caution, particularly where heterogeneity was present. Publication bias was assessed using Egger's regression test, which is commonly applied to detect small-study effects in meta-analyses of observational studies. Egger's test was selected because it provides a quantitative assessment of funnel plot asymmetry and is suitable for continuous effect estimates such as odds ratios and relative risks. Consistent with methodological recommendations, publication bias testing was not performed when fewer than three studies (k < 3) contributed to a pooled estimate, as statistical tests for asymmetry are unreliable and underpowered in such cases. Where applicable, results of publication bias assessments were summarised across risk factors to enhance interpretability.

#### 3.9.2 Epidemiological analysis

A range of quantitative analyses and evaluations were conducted to support risk stratification and identify multiple causal pathways to hypertension, including estimation of the causality index, the critical risk threshold (CRT), and public health importance, as outlined below.

##### 3.9.2.1 Risk factor classification on the basis of the causality index

Hypertension risk factors were grouped into three categories based on Nweke's causality index, as previously reported (15). First-class risk factors, defined at the first quartile of the non-cumulative risk weight (Rw), demonstrated the strongest and most consistent associations with hypertension and warranted immediate intervention. Second-class risk factors, located within the second to third quartiles, contributed to hypertension, but their effects were context-dependent and varied with co-exposures. Third-class risk factors, positioned beyond the third quartile, also participated in hypertension development, but their direct causal influence was weaker compared with first-class risk factors.

##### 3.9.2.2 Determination of the critical risk threshold and modelling hypertension

To identify a causal pathway for hypertension and develop a subsequent multipronged intervention strategy, the critical risk threshold (CRT) was set at the 50th percentile of the cumulative risk weight (15). After the CRT was established**,** the critical risk factors (CRFs), defined as the essential risk factors requiring management to prevent disease at the population level, were determined (15, 18). Quantitatively, CRFs were defined as factors for which the summed risk weights equalled the CRT, provided that each factor fell within the high-moderate to high-priority category. Alternatively, the CRF was conceptualised as a universal set of necessary and component causes. Based on the CRT, hypertension was modelled using different combinations of risk factors (15).

###### 3.9.2.2.1 Operational interpretation of the cumulative risk framework and CRT

To enhance interpretability, the cumulative risk framework operated in three sequential steps. First, pooled effect sizes (ORs) from the meta-analysis were converted into standardised risk weights (Rw) using the expression *Rw* *=* *OR* *×* *Ri*, where *Ri* represented risk responsiveness or consistency of effect. The Rw reflected the relative strength and consistency with which each risk factor was associated with hypertension. Second, these weights were cumulatively summed across combinations of risk factors to generate a cumulative risk weight (Crw). Third, percentile-based thresholds of the Crw distribution were used to define the Critical Risk Threshold (CRT) (15).

In this review, the 50th percentile (CRT = 3.8) represented the point at which population-level preventive strategies became warranted, while the 75th percentile (CRT = 5.5) identified higher-risk constellations appropriate for targeted screening and intensified clinical prevention (15). Importantly, the CRT was not intended as an individual diagnostic cutoff but served as a programmatic planning tool designed to guide prioritisation of prevention strategies rather than predict individual outcomes.

For example, a public health programme focusing on adults aged ≥50 years (Rw = 1.85) with diabetes (Rw = 2.58) yielded a cumulative risk weight of 4.43, exceeding the population-level CRT (3.8) and indicating the need for systematic screening. Inclusion of ethnicity (Rw = 1.57) further increased the cumulative risk to 6.0, exceeding the higher CRT (5.5) and supporting targeted, resource-intensive interventions such as integrated diabetes–hypertension clinics.

By contrast, a public health programme focusing on adults from a mixed-race population (Rw = 1.57), with low socioeconomic status (Rw = 0.38), and lower educational attainment (Rw = 1.75) yielded a cumulative risk weight of 3.70, which remained just below the population-level CRT (3.8). In this scenario, the threshold for systematic hypertension screening was not met, indicating that dedicated screening programmes were not warranted on the basis of these three factors alone. Instead, routine opportunistic screening within existing primary care services was considered appropriate, with escalation to systematic screening only if higher-weight risk factors, such as older age or diabetes, were present**.**

##### 3.9.2.3 Hypertension model: necessary causes, interactions and parsimony

Of the different hypertension models constructed, the frequency of risk factors within each model was assessed to determine the likelihood of necessary causes (15). In addition, hypothetical interactions among predictors were examined using directed acyclic diagrams (DAGs). Although a model was defined as any combination of risk factors whose summed risk weights equaled the CRT, preference was given to models comprising an optimal set of risk factors with the lowest possible interaction density.

### 3.10 Grading the strength of evidence

To evaluate the strength and dependability of the results, the Grading of Recommendations, Assessment, Development, and Evaluation (GRADE) framework was used (27, 30–33). The certainty of evidence was assessed across five principal domains: risk of bias, inconsistency, indirectness, imprecision, and publication bias. This assessment provided a structured method for determining the level of confidence in the collective evidence. By employing systematic grading, this review ensured that recommendations were grounded in evidence-based insights, thereby enhancing hypertension risk stratification strategies.

## 4 Results

### 4.1 Study selection

A total of 43,297 articles were retrieved, and 8,525 duplicates removed. The remaining 34,772 articles were screened by title and abstract; 34,750 ineligible articles were excluded. Full-text screening was performed in 22 studies, of which 11 met inclusion criteria (Figure 1). Twenty factors were identified, with three factors [age, gender and body mass index (BMI)] reported in six studies. Two factors (socioeconomic status and diabetes/blood glucose) were reported in three studies. Two factors (Single nucleotide polymorphism and ethnicity) were reported in two studies, while ten factors (social support, alcohol use, plasminogen activator inhibitor-2, intra-partner violence, exposure to non-partner sexual violence, use of table-added salt, physical activity, employment status, depression and sleep quality.

### 4.2 Study characteristics

The research included 11 studies (three cohort, three case-control, and five nested cross-sectional studies), with 49,058 participants across nine provinces. Gauteng Province hosted two studies (34, 35), as did Eastern Cape Province (36, 37). Western Cape (38) and KwaZulu-Natal (39) each had one study. Five studies used national samples covering nine provinces (40–43) and four provinces (44) (Table 1).

**Table 1:** Study and demographic characteristics.

Authors & Year	Hypertension Definition	Age	Sex	Study Design	Sample size/population/Setting	Province	Race/Ethnicity	Key Outcomes	Notable Predictors/Associations
George et al. (38)	SBP ≥140 mmHg or DBP ≥90 mmHg, or antihypertensive medication use at follow-up	29–53 years	All female	Prospective cohort	478 non-hypertensive SA women, urban	Western Cape Province (Cape Town)	Black South African	57.1% cumulative incidence of hypertension over 8.3 years	Baseline BP strongest predictor; central adiposity more predictive than total fat; DXA did not outperform anthropometry
Naidoo et al. (34)	Elevated BP defined by standardized measurement at age 23; linked to earlier BP trajectories (exact cut-off not explicitly stated in abstract)	23 years	49% male, 51% female	Longitudinal birth cohort (BT20)	1,540 participants aged 23, Soweto	Gauteng Province (Soweto)	Predominantly Black South African	36% had elevated BP at age 23	Higher early-life linear growth ↑ risk; high childhood/adolescent BP trajectories → 4–5× increased adult risk
Ware et al. (40)	SBP ≥140 mmHg or DBP ≥90 mmHg or self-reported diagnosis	18–60+ years (median 51)	68% female	Nested cross-sectional, WHO-SAGE Wave 2	1,847 adults, national SA sample	National sample (all nine provinces)	Mixed South African population (all major ethnic groups)	43% hypertensive; 58% unaware; 33% on meds; 18% controlled	Waist-to-height ratio >0.5 & diabetes strongest predictors; women more aware & treated; lower salt use linked to treatment
Chidumwa et al. (41)	High BP on measurement (cut-off not detailed) or previous diagnosis	Median 56 years	Not specified	Nested cross-sectional spatial modeling	2,761 adults, SA WHO-SAGE Wave 2	National sample (all nine provinces; highest in KwaZulu-Natal & Gauteng)	Mixed South African population (all major ethnic groups)	23.2% high BP; 12.3% diabetes	Hypertension & diabetes co-occur; higher shared spatial risk in KwaZulu-Natal & central Gauteng
Renta et al. (43)	Self-reported and measured hypertension; measured ≥140/90 mmHg	Adult population (exact age range not stated)	Not specified	Nested cross-sectional, WHO-SAGE	4,227	National sample (all nine provinces)	All provinces	Low social capital linked to higher measured hypertension in Ghana but not SA	No rural/urban interaction; country differences in social capital-hypertension link
Patel et al. (44)	BP ≥140/90 mmHg or on medication	20–79 years	Both sexes	Multi-country nested cross-sectional	1,088	Western Cape	Western Cape ethnicity	Central obesity > general obesity prevalence; both predict hypertension & diabetes	In SA, BMI & waist circumference associated with hypertension, but weaker than in other regions
Jacobs et al. (42)	SBP ≥ 140 mmHg or DBP ≥ 90 mmHg, or self-reported antihypertensive medication use	≥15 years (mean 39.7)	65% female	Cohort	3,840 adults, national SA sample	National sample (all nine provinces)	Mixed South African population (all major ethnic groups)	Age-standardised hypertension prevalence = 46%; 53% aware, 40% treated, 16% controlled	Plasminogen activator inhibitor-2 increased hypertension odds by 4% (OR = 1.04, 95% CI: 1.01–1.08).
Mabhida et al. (36)	AHA: SBP ≥130 mmHg or DBP ≥80 mmHg	Adult population (exact age not stated)	Both sexes	Case–control genetic study	442 indigenous SA adults (Mthatha)	Eastern Cape Province (Mthatha)	Indigenous Black South African (Xhosa)	No association between MTHFR rs1801133 and hypertension	Significant interaction between genotype, age & gender
Nguyen et al. (35)	BP ≥140/90 mmHg or previous diagnosis	18–40 years	All female	Nested cross-sectional (Rape Impact Cohort)	1,742 SA women, aged 18–40	Gauteng Province (Johannesburg)	Predominantly Black South African	Hypertension more prevalent in women with IPV, NPSV, SH history	Frequent NPSV, SH, physical & emotional IPV linked to hypertension; depression, PTSD, binge drinking mediated associations
Sharma et al. (37)	Per SA guidelines (likely ≥140/90 mmHg)	Adult population (exact age not stated)	Both sexes	Case–control genetic study	487 isiXhosa-speaking adults (Eastern Cape)	Eastern Cape Province	Black South African (Xhosa)	No association between AGT polymorphisms (4 SNPs) & hypertension	First genetic study on AGT-hypertension in this group
Nkeh et al. (39)	Daytime DBP >90 mmHg on 24-hour ABPM (off medication)	Adult population (exact age not stated)	Both sexes	Case–control genetic study	298 hypertensives & 278 controls, SA	KwaZulu-Natal Province (Durban)	Black South African	ANP intron 2 variants linked to normal BP; NPRC variant not associated	Possible protective role of ANP variant
Styne et al. (10)	Hypertension Definition: SBP ≥140 mmHg or DBP ≥90 mmHg, or on antihypertensive medication	≥15 years (adults)	Both sexes	Cross-sectional (first SA Demographic & Health Survey	13,802 adults	National (all nine provinces)	Black, White, Indian, Mixed-race	Hypertension prevalence highest among Black urban and mixed-race populations; treatment and control rates were low nationwide	ethnicity (Mixed race & Black urban groups at higher risk); obesity and urban residence also linked to hypertension

### 4.3 Definition of hypertension

Studies used different criteria for defining hypertension. Most studies (35, 38, 40, 42, 44) defined it as systolic blood pressure (SBP) ≥140 mmHg or diastolic blood pressure (DBP) ≥90 mmHg, or current antihypertensive medication use. Mabhida et al. (36) used the 2017 American Heart Association definition (≥130/80 mmHg). Nkeh et al. (39) used 24-h ambulatory monitoring, defining hypertension as mean daytime DBP >90 mmHg. Naidoo et al. (34), Chidumwa et al. (41), and Sharma et al. (37) followed South African guidelines or study protocols without specifying thresholds (Table 1).

### 4.4 Risk of bias

The three case–control studies showed strong methodological quality but lacked matching, scoring 9/10 (moderate risk). The cohort studies scored 10.5/11, indicating low risk, with minor follow-up limitations. Five of six nested cross-sectional studies had low risk; Patel et al. (44) showed unclear measurement and confounder handling. Overall, most studies demonstrated strong methodological quality, supporting the reliability of their findings (Supplementary File 2).

### 4.5 Risk factor association with HTN (narrative synthesis)

#### 4.5.1 Genetic polymorphisms (rs variants)

Two case-control studies assessed the relationship between genetic polymorphisms (rs variants) and hypertension (36, 37). Variants rs1801133, rs2004776, rs3789678, and rs7079 were examined across dominant, co-dominant, recessive, and allelic models. The odds ratios ranged between 0.50 and 1.33, with confidence intervals crossing unity in all comparisons, indicating no significant associations. These results suggest that in the sampled South African populations, these polymorphisms may not play a major role in hypertension susceptibility (Table 2).

**Table 2:** Result of individual studies showing factors associated with hypertension in South Africa.

Factors Study HTN definition rs1801133	Reference category	Conditional category	Effect size	Lower limit	Upper limit	Effect Size type
	CC	Dominant:	0.86	0.35	2.16	OR
	CT	Co-dominant	1.33	0.51	3.48	OR
Sharma et al. (37) HTN: ≥140/90 rs2004776	CC	Dominant:	0.91	0.58	1.43	OR
	CT	Co-dominant	0.87	0.61	1.24	OR
	TT	Recessive	1.25	0.85	1.83	OR
rs3789678	CC	Dominant:	1.05	0.73	1.50	OR
	CT	Co-dominant	1.01	0.71	1.45	OR
	TT	Recessive	0.88	0.54	1.45	OR
rs7079	GG	Dominant:	1.32	0.74	2.37	OR
	GA	Co-dominant	0.50	0.19	1.27	OR
	AA	Recessive	1.01	0.49	2.10	OR
Age						OR
Mabhida et al. (36) HTN: ≥130/80	18–35 years	36–49	1.99	1.20	3.31	OR
√	√	50–64	2.71	1.59	4.60	OR
√	√	≥65	3.67	1.77	7.60	OR
Sharma et al. (37)	–	Continuous	6.649	4.71	9.375	OR
Nkeh et al. (39)	–	Continuous	72,052.9	35,203.02	147,450.01	OR
George et al. (38)	–	Continuous	1.54	1.25	1.90	OR
Chidumwa et al. (41) HTN≥140/90	–	Continuous	1.06	1.05	1.07	OR
Ware et al. (40) HTN: BP ≥140/90 mmHg or on antihypertensive medication	–	Continuous	1.04	1.04	1.05	OR
Gender						OR
Mabhida et al. (36)	Female	Male	1.69	1.03	2.78	OR
Nkeh et al. (39)	Female	Male	0.45	0.32	0.64	OR
Sharma et al. (37)	Male	Female	1.2626	0.76	2.1083	OR
Chidumwa et al. (41)	Male	Female	2.45	1.62	3.71	OR
Naidoo et al. (34)	Male	Female	0.38	0.30	0.48	OR
Ware et al. (40)	Male	Female	1.09	0.90	1.33	OR
Ethnicity						OR
Ware et al. (40)	Black	White	1.09	0.90	1.33	OR
√	√	Mixed-race	1.61	1.00	2.58	OR
√	√	Indian	1.74	1.24	2.43	OR
Steyn et al. (10)	Black	Black urban	1.42	1.19	1.68	OR
√	√	Mixed-race	1.541.19–2.00	1.19	2.00	OR
Socioeconomic status						OR
Chidumwa et al. (41)	Lowest wealth tertile	Middle	0.90	0.60	1.34	OR
√	√	Highest	0.83	0.51	1.35	OR
Naidoo et al. (34)	–	Continuous	0.97	0.92	1.02	OR
Renta et al. (43) (Social capital) e ≥140/90	: High	Low	1.13	0.99	1.28	OR
Government support						OR
Chidumwa et al. (41)	No	Yes	3.77	0	1.6 *×* 10^8^	OR
Education						OR
Chidumwa et al. (41) (Yrs of schooling)	–	Continuous	0.90	0.85	0.95	OR
Chidumwa et al. (41)	Primary	Secondary	1.07	0.83	1.37	OR
	√	Tertiary	0.95	0.6	1.51	OR
Ware et al. (40) Year of schooling	–	Continuous	0.89	0.87	0.92	OR
Smoking status						OR
Mabhida et al. (36)	No	Yes	2.58	1.23	5.42	OR
Ware et al. (40) (Ever used tobacco)	No	Yes	1.04	0.83	1.31	OR
Alcohol						OR
Ware et al. (40)		Yes	1.04	0.83	1.31	OR
BMI						OR
Mabhida et al. (36)Ref: Normal	Normal weight	Overweight	1.68	0.98	2.87	OR
√	√	Obesity	3.16	1.95	5.12	OR
Nkeh et al. (39)	–	Continuous	10,234.39	5534.81	18,927.79	OR
Sharma et al. (37)	–	Continuous	3.25	2.34	4.53	OR
Chidumwa et al. (41) Years of schooling	–	Continuous	1.03	1.01	1.05	OR
George et al. (38)	–	Continuous	1.45	1.17	2.75	OR
Patel et al. (44)	–	Continuous	1.08	1.0	1.19	OR
Diabetes/Blood glucose						OR
Mabhida et al. (36) (Diabetes?)	Normal	Prediabetic (FBS >5.6–6.9)	1.34	0.79	2.26	OR
√	√	Diabetic (FBS >7 mmol/L)	1.39	2.11	5.45	OR
Sharma et al. (37) (Blood glucose)	–	Continuous	1.748	1.26	2.4172	OR
Ware et al. (40) (Diabetes?)	No	Yes	2.01	1.31	3.07	OR
Plasminogen activator inhibitor-2-unit increase						OR
Jacobs et al. (42)		Continuous	1.04	1.01	1.08	OR
Intra-partner violence (IPV)						OR
Nguyen et al. (35)	No	Any form of IPV: Yes	1.88	1.37	2.60	OR
√	√	Sexual	1.86	1.33	2.60	OR
√	√	Physical Yes	1.80	1.34	2.40	OR
√	√	Economic	1.95	1.42	2.69	OR
√	√	Life-time exposure to multiple IPV (1–2types)	1.64	1.15	2.32	OR
Nguyen et al. (35)	√	Life-time exposure to multiple IPV (3–4 types)	2.43	1.66	3.55	OR
Exposure to NPSV						OR
Nguyen et al. (35)	No	Exposure to NPSV	1.74	1.26	2.41	OR
√	√	Frequency of NPSV (once)	1.31	0.86	2.01	OR
√	√	Frequency of NPSV (more than once)	2.53	1.62	3.94	OR
√	√	Sexual harassment (Yes)	3.21	2.11	4.87	OR
Use of table-added salt						OR
Ware et al. (40)	No	Yes	0.85	0.70	1.04	OR
Exercise/physical activity						OR
Ware et al. (40)	No	Yes	0.70	0.50	0.98	OR
Currently working						OR
Ware et al. (40)	No	Yes	0.65	0.52	0.82	OR
Depression						OR
Ware et al. (40)	No	Yes	2.10	1.17	3.76	OR
Sleep quality						OR
Ware et al. (40)	Very good/good	Moderate	1.09	0.83	1.44	OR
	√	Poor/very poor	1.21	0.67	2.18	OR

#### 4.5.2 Age

Six studies investigated age as a risk factor for hypertension (36–41). Five studies found positive associations, showing risk increases with age. Mabhida et al. (36) reported that individuals aged ≥65 years had threefold higher odds of hypertension compared to those aged 18–35 (OR = 3.67, 95% CI: 1.77–7.60). Continuous models showed increased odds per unit increase in age, with estimates ranging from OR = 1.04–6.65. One nested cross-sectional study (40) found the association marginal (Table 2).

#### 4.5.3 Gender

Six studies examined gender differences in hypertension risk (34, 36, 37, 39–41). Three studies reported significant associations, with females showing higher odds in some cohorts (e.g., OR = 2.45, 95% CI: 1.62–3.71) (41) and males in others (e.g., OR = 1.69, 95% CI: 1.03–2.78) (36). Naidoo et al. (34) reported a protective effect for females (OR = 0.38, 95% CI: 0.30–0.48) (Table 2).

#### 4.5.4 Ethnicity

Two cross-sectional studies assessed ethnicity (10, 40). Both reported significant associations, with elevated odds among mixed-race and Indian groups in Ware et al. (40) and among black urban and mixed-race groups in Steyn et al. (10). These findings highlight possible sociocultural or environmental factors influencing hypertension prevalence (Table 2).

#### 4.5.5 Socioeconomic status

Three studies explored socioeconomic status (34, 41, 43). None reported significant associations, with ORs close to 1.00 and wide confidence intervals, suggesting that in these populations, socioeconomic status may not independently predict hypertension risk when other factors are considered (Table 2).

#### 4.5.6 Education

Three studies investigated education (40, 41). Two cohort studies found a significant inverse relationship between years of schooling and hypertension (e.g., OR = 0.90, 95% CI: 0.85–0.95), while one cross-sectional study found no association for secondary or tertiary education categories (Table 2).

#### 4.5.7 Smoking status

Two studies (36, 40) examined the topic of smoking. Mabhida et al. (36) reported that current smokers have more than twice the risk of developing hypertension (OR = 2.58, 95% CI: 1.23–5.42). Conversely, Ware et al. (40) found no significant association between having ever used tobacco and hypertension (Table 2).

#### 4.5.8 Body mass Index (BMI)

Six investigations (36–39, 41, 44) consistently indicated that a higher BMI/obesity is a significant risk factor for hypertension. For example, obesity was linked to more than a threefold increase in the odds (OR = 3.16, 95% CI: 1.95–5.12). One study noted no significant link for being overweight, but did find one for obesity (Table 2).

#### 4.5.9 Diabetes/blood glucose

Three studies (36, 37, 40) examined blood glucose levels and identified notable associations between diabetes and prediabetes, with an odds ratio of 2.01 and a 95% confidence interval of 1.31–3.07 for diabetes. Nonetheless, one study reported a non-significant finding for prediabetes (Table 2).

#### 4.5.10 Social support

Findings from a cohort study (41), investigating the role of government support, demonstrated a strong positive correlation with hypertension (OR = 3.77). This could suggest reverse causation, implying that individuals with hypertension might be eligible for financial assistance, rather than the support directly influencing blood pressure (Table 2).

#### 4.5.11 Other factors

Ware et al. (40) reported a non-significant association between alcohol consumption and hypertension (OR = 1.04, 95% CI: 0.83–1.31). Jacobs et al. (42) showed plasminogen activator inhibitor-2 increased hypertension odds by 4% (OR = 1.04, 95% CI: 1.01–1.08). Nguyen et al. (35) identified associations between intimate partner violence (IPV) and hypertension, with odds ratios from 1.64 to 2.43 depending on exposure type. The study found associations between non-partner sexual violence and hypertension, with repeated exposure (OR = 2.53, 95% CI: 1.62–3.94) and sexual harassment (OR = 3.21, 95% CI: 2.11–4.87). Ware et al. (40) reported no significant relationship between table-added salt and hypertension (OR = 0.85, 95% CI: 0.70–1.04), but found physical activity was protective (OR = 0.70, 95% CI: 0.50–0.98) and employment reduced odds (OR = 0.65, 95% CI: 0.52–0.82). Depression linked to higher hypertension odds (OR = 2.10, 95% CI: 1.17–3.76) (40). Sleep quality showed no significant association with hypertension, comparing moderate or poor/very poor with good/very good (40).

### 4.6 Meta-analysis of factors associated with HTN

#### 4.6.1 Age

There was no statistically significant association between age and hypertension (OR = 1.81, 95% CI 0.78–4.22). There was extreme heterogeneity (I^2^ = 99.99%) and publication bias by Egger's test (z = 3.27, *p* = 0.001). Practically, the pooled estimate is unstable because studies vary massively (e.g., one study reported an exceptionally large OR), so individual study estimates—and consistent age definitions—matter more than the overall mean (Table 3; Supplementary File 3).

**Table 3:** Meta-analysis of factors associated with hypertension in South Africa.

Factors	Reference	Odds ratio	95% CI	I^2^ (%)	*p*-value	Risk responsiveness (Ri)	Risk weight (Rw)	Crw	Geo- coverage	Temporal coverage	Geo-temporal trend (GTT)
Age (years)	Per 1 year increase	1.13	1.07–1.20	97.70	<0.001	1.0	1.13	–	–	–	–
√	Per 5 years increase	1.85	1.39–2.46	97.70	<0.001	1.0	1.85^U^	1.85	1.0	0.67	0.67 (Adj: 0.83)
√	Per 10 years increase	3.43	1.92–6.08	97.70	<0.001	1.0	3.43	–	–	–	–
Sex	Female vs. Male	1.09	0.59–2.00	95.40	<0.001	NS	–	–	1.0	1	1.0 (Adj: NA)
SES	Low SES	1.14	1.01–1.30	0.00	0.820	0.33^b^	0.38^L^	2.23	1.0	1/3	0.33 (Adj: 0.33)
BMI	Per 1 unit increase	1.71	0.83–3.49	95.10	<0.001	NS	–	–	0.22	2/3	0.15 (Adj: NA)
Ethnicity	Mixed-race vs. black	1.57	1.23–2.01	0.00	0.8720	1.0	1.57^L^	3.80	1.0	2/3	0.67 (Adj: 0.83)
Education (years of schooling)	Per 1 year increase	0.89 [1.12]^a^	0.87–0.92 [1.09–1.15]	0.00	0.725	1.0	–	–	–	–	–
√	Per 5 years increase	0.57 [1.75]^a^	0.50–0.64 [1.56–2.00]	0.00	0.725	1.0	1.75^M^	5.55	1.0	2/3	0.67 (Adj: 0.83)
√	Per 10 years increase	0.32 [3.13]^a^	0.25–0.41 [2.44–4.00	0.00	0.725	1.0	–	–	–	–	–
Diabetes	Yes	2.58	1.55–4.31	61.32	0.108	1.0	2.58^U^	8.13	1.0	2/3	0.67 (Adj: 0.83)
Smoking	Yes	1.53	0.63–3.68	81.02	0.022	NS	–	–	1.0	2/3	0.67 (Adj: NA)

NS, not statistically significant at *α* = 0.05; NA, the computation of the Adjusted GTT is not useful; Crw, cumulative risk weight; F, First-class factors; S, second-class factors; T, third class factors; U, upper class ≥1.85, M, middle class (1.75–1.84); L, lower class factors (≤1.74).

^a^ Hypothetical risk for reduced (lower) years of schooling.

b When none of the primary studies were significant, the significant pooled OR is counted as though one of the primary studies, and the ratio taken as appropriate.

#### 4.6.2 Diabetes

There was a statistically significant association between diabetes and hypertension (OR = 2.58, 95% CI 1.55–4.31). There was moderate heterogeneity (I^2^ = 61.32%), and publication bias was not assessed (k < 3). Both included studies point to higher odds with diabetes, but with only two studies the uncertainty around between-study differences remain (Table 3; Supplementary File 3).

#### 4.6.3 Education

There was a statistically significant protective association (OR = 0.89, 95% CI 0.87–0.91). No heterogeneity was detected (I^2^ = 0%), and publication bias was not assessed (k < 3). This is a consistent, precise signal across studies—higher education is linked to lower odds of hypertension—likely reflecting socioeconomic advantages and health literacy, though causality can't be inferred from observational data (Table 3; Supplementary File 3).

#### 4.6.4 Ethnicity (mixed-race vs. black)

There was a statistically significant association (OR = 1.56, 95% CI 1.24–1.95). No heterogeneity was detected (I^2^ = 0%), and publication bias was not assessed (k < 3). The effect is consistent across two South African studies (Table 3; Supplementary File 3).

#### 4.6.5 Gender (female vs. male)

There was no statistically significant association (OR = 1.09, 95% CI 0.60–1.96). Very high heterogeneity was present (I^2^ = 95.04%), with no evidence of publication bias (Egger's z = 0.48, *p* = 0.632). The wide between-study spread (some >2, some <1) suggests differences in adjustment sets, age structure, or measurement context—so sex-specific risks likely depend on population and covariates rather than a uniform effect (Table 3; Supplementary File 3).

#### 4.6.6 BMI

The relationship between BMI and hypertension in South African populations shows complexity depending on whether BMI is treated categorically or continuously. Individual studies consistently reported that obesity, compared to normal weight, was associated with more than a threefold increased risk of hypertension [e.g., OR = 3.25, 95% CI: 2.34–4.52 (37); OR = 3.16, 95% CI: 1.95–5.12 (36)]. However, the pooled meta-analysis of BMI as a continuous variable yielded a non-significant overall association (OR = 1.70, 95% CI 0.88–3.28, Egger's z = 0.58, *p* = 0.560), as shown in the forest plot. This discrepancy highlights that the risk of hypertension may rise disproportionately at higher thresholds of BMI (particularly in the obese range), whereas incremental increases in BMI across the full spectrum may not consistently predict risk in the same populations (Table 3; Supplementary File 3).

#### 4.6.7 Socio-economic status (SES)

There was no statistically significant association between SES and hypertension (OR = 0.94, 95% CI 0.86–1.02). Low–moderate heterogeneity was present (I^2^ = 31.51%), with no evidence of publication bias (Egger's z = –1.22, *p* = 0.222). The slight protective trend with higher SES is plausible, but mixing categorical “high vs. low” with continuous SES metrics likely dilutes clarity. Hence, we conducted a subgroup analysis including only two studies with continuous SES metric showed a statistically significant association between socio-economic status and hypertension (OR = 0.88, 95% CI 0.77–0.99). No heterogeneity was present (I^2^ = 0.00%), and publication bias was not assessed (k < 3) (Table 3; Supplementary File 3).

#### 4.6.8 Smoking

There was no statistically significant association (OR = 1.53, 95% CI 0.63–3.68). High heterogeneity was present (I^2^ = 81.02%), and publication bias was not assessed (k < 3). The two studies diverge (one suggests elevated odds, the other near-null) (Table 3; Supplementary File 3).

### 4.7 Epidemiological synthesis

#### 4.7.1 Risk responsiveness and geotemporal trend

Of the five risk factors identified, four [age (Ri = 1.0), ethnicity (Ri = 1.0), education (Ri = 1.0) and diabetes (Ri = 1.0) possessed perfect risk responsiveness, whereas SES possessed a low-risk responsiveness (Ri = 0.33)]. However, it is important to note that the risk responsiveness of these five factors might change as more studies emerge. Four of the five factors (age, ethnicity, education and diabetes) showed a broad geographical coverage, with participants drawn from all nine provinces. The four factors were reported in two of the three study periods (2000–2010, 2011–2020, and ≥2021). Similarly, four of the five factors [age (GTT = 0.83), ethnicity (GTT = 0.83), education (GTT = 0.83), and diabetes (GTT = 0.83)] possessed an acceptable geotemporal coverage (Table 3). SES showed a low geotemporal coverage (GTT = 0.33) (Table 3).

#### 4.7.2 Risk stratification and critical threshold (CRT) for hypertension in South Africa

**Figure 2:**
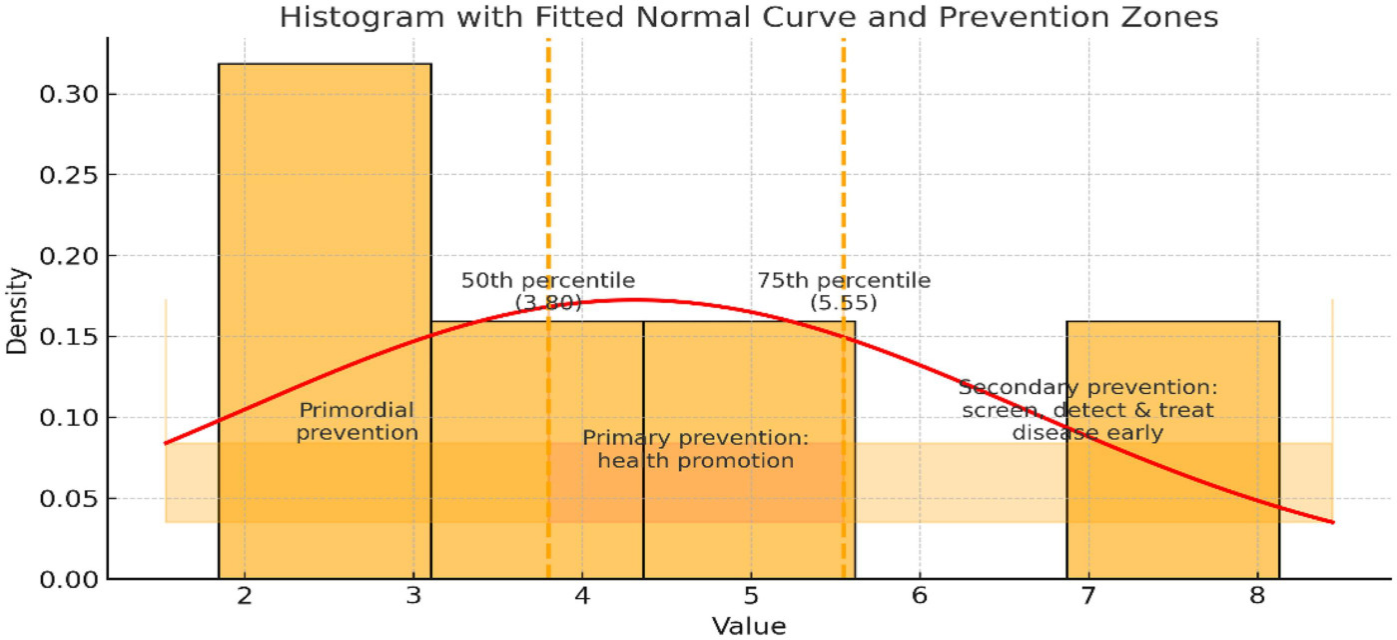
Histogram showing the Rw-based CRT for different levels of prevention.

The Rw was used to classify risk factors. Diabetes (Rw = 2.58) and age (Rw = 1.85) belonged to upper class category. Education belonged to middle class risk factors, whereas ethnicity, ethnicity (Rw = 1.57) and SES (Rw = 0.38) fell into lower class category (Table 3). The critical risk threshold (CRT) was fitted at 3.8 (50th percentile of the cumulative Rw) and 5.5 (75th percentile of the cumulative Rw), corresponding the point of primary and secondary/clinical prevention (Figure 2). Given the limitedness of data included in this synthesis, it is difficult to spell out which of these factors were “necessary causes” or “component causes”. However, age (Rw = 1.85, GTT = 0.83) and diabetes (Rw = 2.58, GTT = 0.83) appear to be the most indispensable risk factors of hypertension in South Africa (Tables 3, 4). We assumed that hypertension is imminent in individuals whose risk factor combination equals the CRT. In addition, factors with a GTT of 0.6 were preferred to improve the utility of the emergent model. A GTT of 0.6 signifies fair geographical (5/9 provinces) and temporal coverage (2/3 periods) (Table 3).

**Table 4:** Hypertension prediction from risk factors and checklist combinations.

Model/causal pathway	k (size)	Risk Factors Combination	Threshold (Summative Risk Weight)	Summative GTT	Interaction Count	Interaction Density
1	3	Age, Ethnicity, Diabetes	6.0	2.49	2	0.667
2	3	Age, Education, Diabetes	6.18	2.49	3	1.0
3	3	Ethnicity, Education, Diabetes	5.9	2.49	3	1.0
4	4	Age, SES, Ethnicity, Education	5.55	2.82	5	0.833
5	4	Age, SES, Ethnicity, Diabetes	6.38	2.82	5	0.833
6	4	Age, SES, Education, Diabetes	6.56	2.82	5	0.833
7	4	Age, Ethnicity, Education, Diabetes	7.75	2.82	6	1.0
8	4	SES, Ethnicity, Education, Diabetes	6.28	3.32	6	1.0
9	5	Age, SES, Ethnicity, Education, Diabetes	8.13	3.65	9	0.9

#### 4.7.3 Modelling hypertension in South Africa: causal paths, interaction and parsimony

**Figure 3:**
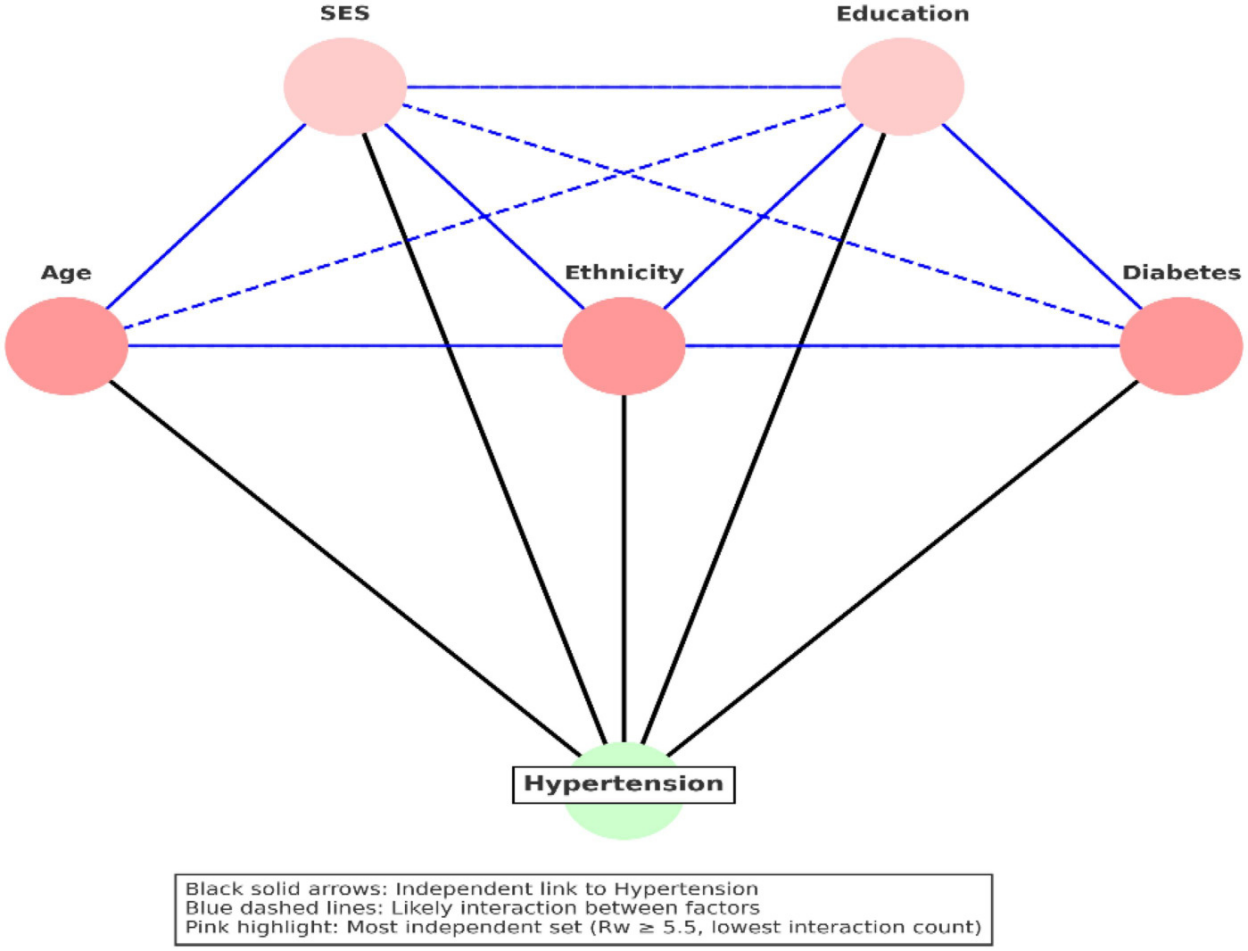
DAGs showing the hypothetical interaction between predictors of hypertension in South Africa.

There were nine possible ways in which the five risk factors could combine to reach the CRT (Table 4). Upon examination of the hypothetical interaction among the five putative risk factors of hypertension in South Africa, result shows there were nine models (causal paths) through which these factors may interact to induce hypertension, with the Age─Ethnicity─Diabetes being the most parsimonic model with minimal interaction density (Table 4). The optimum model of hypertension in South Africa could be any of Age─Ethnicity─Diabetes, Age─SES─Ethnicity─Diabetes─Age─SES─Education, Diabetes or Age─SES─Ethnicity─Education─Diabetes (Table 4, Figure 3).

#### 4.7.4 Causality index and public health priority

The evidence underpinning the meta-synthesis of most (four of five) risk factors was very limited hence we did not undertake casualty and public health priority as this may result in a misleading finding. We expect to carry out this analysis in future reviews as more primary studies emerge.

### 4.8 Certainty of evidence

This review assessed common risk factors for high blood pressure (hypertension) among adults in South Africa. The evidence shows that older age, belonging to certain mixed-race group, and having diabetes substantially increase the chance of developing hypertension. Higher body mass index (BMI) may also raise risk, but the evidence is less consistent. On the other hand, more years of schooling clearly reduce the risk, highlighting the protective effect of education. Socio-economic status (SES) appears to play a small role, while the effects of sex (male vs. female) and smoking remain uncertain because the available studies were small and inconsistent. Overall, the strongest and most reliable risk factors identified were age, ethnicity, diabetes, and education. These findings suggest that public health interventions should focus on early screening of high-risk groups, promoting diabetes control, and expanding access to education as a long-term preventive measure (Table 5).

**Table 5:** Certainty of evidence for each risk factor underscoring the review findings.

Risk factor (Population: Adults in South Africa)	Effect (OR, 95% CI)	No. of studies	Certainty of evidence (GRADE)	Reasons for downgrade/upgrade	Interpretation
Age	1.85 (1.39–2.46)	3	⊕⊕⊕⦻ Moderate	Publication bias -1	Age is moderately associated with increased hypertension risk.
Sex	1.09 (0.59–2.00)	6	⊕⊕⦻⦻ Low	Imprecision -1; Inconsistency -1	No consistent association; evidence uncertain.
Socio-economic status (SES)	1.14 (1.01–1.30)	2	⊕⊕⊕⦻ Moderate	Inconsistency -1	Slight increase in risk, though findings vary.
BMI	1.71 (0.83–3.49)	3	⊕⊕⦻⦻ Low	Imprecision -1; Inconsistency -1	Evidence suggests risk but is inconsistent and imprecise.
Ethnicity	1.57 (1.23–2.01)	2	⊕⊕⊕⊕ High	None	Strong evidence of increased risk by ethnicity.
Education	0.57 (0.50–0.64)	2	⊕⊕⊕⊕ High	None	Higher education is protective against hypertension.
Diabetes	2.58 (1.55–4.31)	2	⊕⊕⊕⦻ Moderate	Imprecision -1; Inconsistency -1; Large effect +1	Diabetes strongly increases hypertension risk.
Smoking	1.53 (0.63–3.68)	2	⊕⊕⦻⦻ Low	Imprecision -1; Inconsistency -1	Evidence uncertain; effect may exist but imprecise.

## 5 Discussion

This systematic review and meta-analysis synthesized evidence on the risk factors for hypertension in South Africa, integrating epidemiological models to enhance risk classification and prediction. The findings identified both traditional and social factors with varying levels of predictive strength. Advanced age and diabetes emerged as consistent and necessary causes, whereas socio-economic status, years of schooling and ethnicity as component or synergistic factors. By applying structured metrics—such as risk weight, risk responsiveness, and geotemporal coverage—this preliminary study offers a refined framework for understanding hypertension risk in South Africa, with implications for clinical decision-making, public health policy, and future research.

### 5.1 Age

In this study, age was linked to increased hypertension risk in South African studies, with older adults (≥65 years) having nearly fourfold higher odds compared to those aged 18–35 (36). This aligns with national and global evidence showing a rise in hypertension prevalence with age, although South Africans develop hypertension earlier and with lower awareness compared to high-income settings (41, 45). The biological basis is well established: ageing causes arterial stiffening, endothelial dysfunction, oxidative stress, and neurohormonal changes that elevate blood pressure (46, 47). Age is also a frequent predictor in hypertension risk models, like the Framingham Hypertension Risk Score and other global algorithms, because it improves predictive accuracy (48, 49). Although non-modifiable, age is crucial for primary prevention as it helps stratify populations for earlier screening and intervention, especially in South Africa, where hypertension onset occurs younger.

### 5.2 Ethnicity

Ethnicity influences hypertension risk in South Africa, with the mixed-race population as the highest-risk group. Ware et al. (40) reported higher hypertension odds among mixed-race adults vs. black and white counterparts, while Steyn et al. (10) highlighted higher prevalence in urban black and mixed-race populations, with the mixed-race group most vulnerable. Global reviews show ethnic disparities in hypertension reflect interactions between genetic predisposition, salt sensitivity, stress, and socio-economic disadvantage (50, 51). Mixed-race populations may be more susceptible due to combined African and European ancestry–related vascular traits, compounded by obesity, diabetes, and lifestyle factors (52). Ethnicity is non-modifiable, but its predictive value is recognized in international hypertension risk algorithms (e.g., QRISK, Framingham extensions). From a public health perspective, interventions cannot alter ethnicity, but can target modifiable factors in mixed-race communities—such as obesity, diet, stress, and healthcare access—making tailored strategies critical.

### 5.3 Socioeconomic status

Although socioeconomic status is often conceptualized as a distal or contextual determinant, the present findings reinforce its relevance as a clinically meaningful predictor of hypertension in South Africa. Although categorical measures of socioeconomic status or wealth index were not significantly associated with hypertension in the pooled analysis (34, 41, 43), the continuous SES subgroup showed a modest but statistically significant protective effect, with higher SES associated with lower hypertension risk. This discrepancy underscores the influence of how SES is operationalised in epidemiological analyses. Broad categorical groupings may obscure gradient or dose–response relationships, whereas continuous measures are more sensitive to incremental socioeconomic differences. In settings characterised by marked socioeconomic heterogeneity, such as South Africa, the use of continuous or composite SES indicators may therefore provide more informative estimates for hypertension risk stratification and public health planning.

Nonetheless, this finding is consistent with international evidence linking low SES to elevated hypertension risk through pathways involving chronic stress, poor diet quality, limited healthcare access, and adverse physical environments (53, 54).

Importantly, the relationship between SES and hypertension is biologically mediated. Chronic exposure to socioeconomic adversity activates the hypothalamic–pituitary–adrenal axis and the sympathetic nervous system, leading to sustained elevations in cortisol and catecholamines. These neuroendocrine responses promote endothelial dysfunction, increased vascular resistance, sodium retention, and activation of the renin–angiotensin–aldosterone system, all of which contribute directly to blood pressure elevation (55). From a clinical perspective, low SES also constrains effective hypertension management through delayed healthcare utilisation, inconsistent medication access, and reduced continuity of care. Although traditional risk prediction tools such as the Framingham Risk Score and WHO/ISH charts do not explicitly include SES, emerging cardiovascular frameworks increasingly recognise its role in shaping both biological risk and treatment outcomes. As a modifiable structural determinant, SES represents a critical target for policy-driven hypertension prevention in South Africa.

### 5.4 Education status

Education emerged as an important social determinant of hypertension in South Africa. Two cohort studies demonstrated an inverse association between years of schooling and hypertension risk, while one cross-sectional study reported no significant association (40, 41). These findings are consistent with broader epidemiological evidence showing that lower educational attainment is associated with increased hypertension and cardiovascular risk, particularly in low- and middle-income settings.

The association between education and hypertension is biologically plausible. Lower educational attainment is frequently linked to chronic psychosocial stress, reduced occupational control, and limited coping resources, which activate the hypothalamic–pituitary–adrenal axis and the sympathetic nervous system. Sustained elevations in cortisol and catecholamines promote endothelial dysfunction, vascular stiffness, sodium retention, and dysregulation of the renin–angiotensin–aldosterone system, all of which contribute to persistent blood pressure elevation (55–57).

From a clinical perspective, education influences hypertension risk and outcomes through health literacy, healthcare utilisation, and adherence to antihypertensive therapy. Individuals with lower education may have difficulty understanding hypertension diagnosis and the need for long-term treatment, resulting in delayed care-seeking and poorer blood pressure control, even after treatment initiation (58) Although education is not explicitly included in major hypertension prediction tools such as the WHO/ISH charts or Framingham Risk Score, its effects are indirectly captured through socioeconomic and behavioural correlates. As a modifiable upstream determinant, improving educational attainment and targeted health education may reduce hypertension risk and improve clinical outcomes in South Africa.

### 5.5 BMI

The association between BMI and hypertension varies by context. South African studies reported strong associations between obesity and hypertension (36, 37), while pooled meta-analysis of BMI as a continuous variable showed a non-significant relationship (OR = 1.49, 95% CI: 0.89–2.50). This suggests hypertension risk increases disproportionately at higher BMI thresholds, while incremental BMI increases may not consistently predict risk. Systematic reviews confirm obesity as a major determinant of hypertension, though effects are weaker in some sub-Saharan African cohorts (59, 60). Excess adiposity elevates blood pressure through renin–angiotensin–aldosterone system activation, heightened sympathetic drive, and endothelial dysfunction. Predictive tools like Framingham Risk Score and WHO/ISH charts include BMI, though waist circumference may be more predictive in African contexts. As a modifiable risk factor, BMI remains relevant for primary prevention, with obesity interventions offering potential for reducing hypertension in African populations.

### 5.6 Diabetes

Diabetes has consistently been shown to be a strong predictor of hypertension in South African studies (38, 41) and globally, where it approximately doubles the risk (51, 61). The biological plausibility rests on mechanisms such as insulin resistance, sodium retention, endothelial dysfunction, and vascular remodeling, all of which elevate blood pressure. Predictive algorithms like the Framingham Risk Score and WHO/ISH cardiovascular charts routinely include diabetes, underscoring its predictive value. While not entirely modifiable, effective glycemic control and lifestyle interventions can reduce hypertension risk, making diabetes prevention and management critical for primary prevention strategies in African settings facing rising metabolic disease burdens.

### 5.7 Smoking

Smoking's link to hypertension in South Africa is inconsistent. Mabhida et al. (36) found smokers had twice the odds of hypertension (OR = 2.58, 95% CI: 1.23–5.42), but Ware et al. (40) noted no significant link for tobacco users. This mirrors global reviews where smoking is a cardiovascular risk factor, but its independent effect on hypertension is modest after adjusting for BMI, alcohol, and age (51). None of the studies adjusted for these factors. Nicotine biologically increases sympathetic activity, causes vasoconstriction, and impairs endothelial function, raising blood pressure. Predictive models like the Framingham Risk Score and SCORE2 exclude smoking as a direct hypertension predictor but include it for cardiovascular disease, underscoring its broader vascular risk. Smoking is a modifiable risk factor, and cessation programs are key in prevention, reducing cardiovascular morbidity and hypertension risk.

### 5.8 Implications for practice and policy

The findings from this review indicate that risk weight (Rw) scoring provides a practical approach for stratifying hypertension risk in South Africa, with a cumulative threshold of 5.5 identifying populations that warrant targeted screening. Older adults (≥50 years), individuals with diabetes, mixed-race populations, and those from lower socioeconomic strata emerge as priority surveillance groups (10, 36, 41). These groups account for combinations of risk factors that consistently exceed the critical risk threshold, supporting focused rather than universal screening strategies.

In practical terms, adults aged ≥50 years with diabetes represent a high-priority group for systematic hypertension screening, particularly within mixed-race communities where cumulative risk exceeds the higher CRT. For this group, blood pressure screening should be routine at every clinical encounter, embedded within diabetes care pathways, and supported by integrated management models that address shared cardiometabolic risk. This “opportunistic-plus” approach strengthens early detection while remaining feasible within resource-constrained health systems.

At a broader prevention and policy level, the findings highlight the importance of addressing modifiable upstream determinants alongside clinical screening. Improving educational attainment (40), reducing socioeconomic disadvantage through employment and social protection initiatives (34, 43), and ensuring effective glycaemic control among people with diabetes (37, 38) are likely to yield downstream benefits for hypertension prevention. Collectively, these findings support the integration of risk-weighted approaches into early detection, service planning, and prevention programmes, in alignment with WHO recommendations and South African national hypertension control strategies (60, 62).

### 5.9 Actionable recommendations

First, at the screening level, we recommend routine blood pressure screening for adults aged ≥50 years with diabetes at every clinical encounter, regardless of presenting complaint, with lower thresholds for follow-up in mixed-race populations where risk accumulation exceeds the higher CRT.
Second, at the service-delivery level, we propose integrated diabetes–hypertension care pathways, including combined clinic visits, shared risk counselling, and synchronized medication reviews, to address the high co-occurrence and shared pathophysiology of these conditions.
Third, at the community and policy level, we emphasize prioritizing outreach and preventive programmes in populations where age and diabetes co-exist, supported by task-shifting to primary care and community health workers to improve early detection and continuity of care.

## 6 Strengths and limitations

The strength of this study lies in its use of relevant theoretical reasoning to assemble a causality framework and determine the critical risk threshold (CRT), thereby offering clinical decision support. However, several limitations should be noted. First, the internal metrics used—risk weight (Rw), confidence intervals (CI), and probability of hypertension prediction (PHP)—require external validation before widespread application. Second, heterogeneity in the definition and measurement of explanatory and outcome variables across included studies introduced methodological variability. Third, the absence of formal sensitivity analyses reflects the limited number of studies available for several pooled estimates and should be considered when interpreting the robustness of the findings. Fourth, the review protocol employed innovative principles that have not yet been subjected to formal quantitative validation, which may limit its robustness. Finally, the relatively small number of eligible studies, resulting from the strict selection criteria, reduces the external validity of the findings and limits their generalizability.

## 7 Conclusion

Although age and diabetes consistently appeared across high-risk combinations, the limited number of included studies precludes definitive causal classification. These factors are therefore interpreted as core contributors within the current evidence base, rather than necessary causes in a strict causal sense. These findings highlight the need for causal reasoning in risk stratification to strengthen clinical and public health assessments. Notably, dietary, behavioural, and socioeconomic exposures were poorly sampled in eligible studies, limiting interpretation. Future research should apply robust epidemiological frameworks to improve variable coverage and enhance predictive modelling for both prevention and clinical care.
