# Supplementary material for: Risk stratification of hypertension in South Africa: a systematic review with meta-analysis
Source: Front Cardiovasc Med. 2026 Feb 23;13:1710798. doi: 10.3389/fcvm.2026.1710798 (PMC12967931; doi:10.3389/fcvm.2026.1710798)
Supplement: Supplementary file 1 [file Table1.docx]

# Supplementary File 1: Assessment of publication bias for hypertension risk factors

| Risk factor | Number of studies (k) | Egger’s test performed | Egger’s z value | p-value | Interpretation |
| --- | --- | --- | --- | --- | --- |
| Age | ≥3 | Yes | 3.27 | 0.001 | Evidence of publication bias; pooled estimate unstable |
| Diabetes | 2 | No | Not performed | Not performed | Egger’s test not assessed (k < 3) |
| Education | 2 | No | Not performed | Not performed | Egger’s test not assessed (k < 3) |
| Ethnicity (mixed-race vs Black) | 2 | No | Not performed | Not performed | Egger’s test not assessed (k < 3) |
| Sex (female vs male) | ≥3 | Yes | 0.48 | 0.632 | No evidence of publication bias |
| BMI (continuous) | ≥3 | Yes | 0.58 | 0.560 | No evidence of publication bias |
| Socio-economic status (overall) | ≥3 | Yes | -1.22 | 0.222 | No evidence of publication bias |
| Socio-economic status (continuous subgroup) | 2 | No | Not performed | Not performed | Egger’s test not assessed (k < 3) |
| Smoking | 2 | No | Not performed | Not performed | Egger’s test not assessed (k < 3) |
