# Supplementary material for: Risk stratification of hypertension in South Africa: a systematic review with meta-analysis
Source: Front Cardiovasc Med. 2026 Feb 23;13:1710798. doi: 10.3389/fcvm.2026.1710798 (PMC12967931; doi:10.3389/fcvm.2026.1710798)
Supplement: Supplementary file 2 [file Datasheet1.docx]

*SEARCH STRATEGY*

*PubMed (No retrieved:* ***9262; no filter applied)***

((Hypertension or high blood pressure or elevated blood pressure or htn or hypertensive or blood pressure [MeSH Terms]) AND (Risk factors or correlates OR determinants OR predictors OR contributing factors OR predisposing factors OR causes OR vulnerability factors OR associate*)) AND (sub-Saharan Africa OR Africa*[MeSH Terms])

***Academic search complete (****(No retrieved:* ***9904; no filter applied)***

Hypertension or high blood pressure or elevated blood pressure or htn or hypertensive or blood pressure [SU] AND Risk factors or correlates OR determinants OR predictors OR contributing factors OR predisposing factors OR causes OR vulnerability factors OR associate* [SU] AND sub-Saharan Africa OR Africa OR Botswana OR Burkina Faso OR Burundi OR Cameroon OR Cape Verd OR Central African Republic OR Chad OR Comoros OR Congo OR Rep OR Congo OR Cote d'Ivoire OR Equatorial Guinea OR Eritrea OR Ethiopia OR Gabon OR Ghana OR Guinea OR Guinea-Bissau OR Kenya OR Lesotho OR Liberia OR Madagascar OR Malawi OR Mali OR Mauritania OR Mauritius OR Mozambique OR Namibia OR Niger OR Nigeria [SU]

*CINAHL (No retrieved:* ***3,082 ; no filter applied)***

Hypertension or high blood pressure or elevated blood pressure or htn or hypertensive or blood pressure [SU] AND Risk factors or correlates OR determinants OR predictors OR contributing factors OR predisposing factors OR causes OR vulnerability factors OR associate* [SU] AND sub-Saharan Africa OR Africa OR Botswana OR Burkina Faso OR Burundi OR Cameroon OR Cape Verd OR Central African Republic OR Chad OR Comoros OR Congo OR Rep OR Congo OR Cote d'Ivoire OR Equatorial Guinea OR Eritrea OR Ethiopia OR Gabon OR Ghana OR Guinea OR Guinea-Bissau OR Kenya OR Lesotho OR Liberia OR Madagascar OR Malawi OR Mali OR Mauritania OR Mauritius OR Mozambique OR Namibia OR Niger OR Nigeria [SU]

*MEDLINE (No retrived* ***8,685, No filter applied)***

Hypertension or high blood pressure or elevated blood pressure or htn or hypertensive or blood pressure [SU] AND Risk factors or correlates OR determinants OR predictors OR contributing factors OR predisposing factors OR causes OR vulnerability factors OR associate* [SU] AND sub-Saharan Africa OR Africa OR Botswana OR Burkina Faso OR Burundi OR Cameroon OR Cape Verd OR Central African Republic OR Chad OR Comoros OR Congo OR Rep OR Congo OR Cote d'Ivoire OR Equatorial Guinea OR Eritrea OR Ethiopia OR Gabon OR Ghana OR Guinea OR Guinea-Bissau OR Kenya OR Lesotho OR Liberia OR Madagascar OR Malawi OR Mali OR Mauritania OR Mauritius OR Mozambique OR Namibia OR Niger OR Nigeria [SU]
